# Convolutional neural networks combined with conventional filtering to semantically segment plant roots in rapidly scanned X-ray computed tomography volumes with high noise levels

**DOI:** 10.1186/s13007-024-01208-0

**Published:** 2024-05-21

**Authors:** Shota Teramoto, Yusaku Uga

**Affiliations:** Institute of Crop Sciences, National Agriculture & Food Research Organization, Tsukuba, Ibaraki 305-8602 Japan

**Keywords:** Root system architecture, RSA, X-ray CT, 3D, Semantic segmentation, Deep learning, Convolutional neural network, Edge detection, Image processing, Rice

## Abstract

**Background:**

X-ray computed tomography (CT) is a powerful tool for measuring plant root growth in soil. However, a rapid scan with larger pots, which is required for throughput-prioritized crop breeding, results in high noise levels, low resolution, and blurred root segments in the CT volumes. Moreover, while plant root segmentation is essential for root quantification, detailed conditional studies on segmenting noisy root segments are scarce. The present study aimed to investigate the effects of scanning time and deep learning-based restoration of image quality on semantic segmentation of blurry rice (*Oryza sativa*) root segments in CT volumes.

**Results:**

VoxResNet, a convolutional neural network-based voxel-wise residual network, was used as the segmentation model. The training efficiency of the model was compared using CT volumes obtained at scan times of 33, 66, 150, 300, and 600 s. The learning efficiencies of the samples were similar, except for scan times of 33 and 66 s. In addition, The noise levels of predicted volumes differd among scanning conditions, indicating that the noise level of a scan time ≥ 150 s does not affect the model training efficiency. Conventional filtering methods, such as median filtering and edge detection, increased the training efficiency by approximately 10% under any conditions. However, the training efficiency of 33 and 66 s-scanned samples remained relatively low. We concluded that scan time must be at least 150 s to not affect segmentation. Finally, we constructed a semantic segmentation model for 150 s-scanned CT volumes, for which the Dice loss reached 0.093. This model could not predict the lateral roots, which were not included in the training data. This limitation will be addressed by preparing appropriate training data.

**Conclusions:**

A semantic segmentation model can be constructed even with rapidly scanned CT volumes with high noise levels. Given that scanning times ≥ 150 s did not affect the segmentation results, this technique holds promise for rapid and low-dose scanning. This study offers insights into images other than CT volumes with high noise levels that are challenging to determine when annotating.

**Supplementary Information:**

The online version contains supplementary material available at 10.1186/s13007-024-01208-0.

## Background

Plant root distribution in the soil is a crucial phenotype that determines nutrient and water availability. Generally, nutrient distribution in the soil is greatly influenced by fertilizer application, whereas water distribution is greatly influenced by the weather. Altering root distribution to gain access to soil resources is a practical strategy to increase crop yield. Changes in root distribution in the soil increase yield by enhancing nutrient uptake under irrigated paddy [1] and water uptake under upland conditions with drought [2] in rice (*Oryza sativa*). Therefore, root distribution that maximizes benefits strongly depends on the plant growth environment [3–5]. As the environment changes significantly during the growing period, the optimal root system also changes. For example, under top-dressing fertilization conditions, shallow, wider, and denser root systems are advantageous [6]. In contrast, deeper, narrower, and denser root systems are advantageous under drought conditions as deeper soils contain more water [7–10]. An ideal root system responds to the environment by increasing root density in soil areas with fertilizer and sufficient moisture. Therefore, these effects of changes in the root system must be evaluated in an integrated, time-series manner to improve crop root morphology.

Non-destructive measurements can help evaluate root growth dynamics over time. In particular, X-ray computed tomography (CT) and magnetic resonance imaging, which can measure changes in root distribution in three dimensions (3D), are powerful tools for assessing changes in root distribution in heterogeneous soil environments [11, 12], such as those used for local fertilization [13, 14], local soil compaction [15, 16], and in the field [17–19]. However, 3D volume data are large, and image analyses, such as extracting root segments from the 3D volume, calculating root traits, and analyzing time-series 3D data, are labor-intensive. Therefore, many methods have been developed for efficiently analyzing these 3D volumes. These include methods to isolate root segments by conventional filtering, such as thresholding and edge extraction using uniform soil [20, 21], region growing [22, 23], root tracking [24, 25], root filtering with shape-based filters [26, 27], and efficient analysis of time-series CT volumes [28–30]. However, the conditions under which these methods can be used limit their applicability. Therefore, a versatile analysis method independent of these conditions is required.

Semantic segmentation is a powerful method for extracting regions of interest (ROI) from the image data [31]. Generally, semantic segmentation produces image data in which ROIs are labeled as the foreground and the other regions as the background using convolutional neural networks (CNNs). Therefore, a model can be trained to extract only the ROI from an image with a complex background using appropriate training data. This advantage is also effective in extracting roots from heterozygous soil images. For example, conventional image processing algorithms, such as thresholding and edge detection, detect some backgrounds as false positives unless homozygous soil grains are used [20]. In this case, only complete root information must be extracted using root tracking or other methods [24, 25, 32]. Alternatively, semantic segmentation can be used independently of the background. Recently, semantic segmentation has been frequently used in plant root phenotyping. In 2D, semantic segmentation can extract roots from images captured from the side of a rhizobox, a container with transparent sides to observe roots, and with a mini-rhizotron, an instrument for acquiring an image of a surface by burying it in soil [33–37]. These images often include voids, bubbles, and light reflections on the transparent sides as the background. Moreover, digital images captured outdoors may exhibit changes in the color, brightness, and shadows of the soil. Therefore, incorporating these changes in the training data enables root extraction using semantic segmentation [38].

Semantic segmentation has been used for 3D volume and 2D images. However, 3D segmentation reconstructed from 2D segmentation processed slice-by-slice tends to overlook 3D connections; therefore, a model utilizing 3D images as input is superior [39]. Semantic segmentation in 3D is used to extract roots of various thicknesses [40]. A conventional filtering method, such as shape-based filtering [26, 27, 41], can be used if the root thickness variation is minimal, whereas alternative approaches are required for large variations [40]. This variation can be addressed by adding a spatial pyramid pooling layer [42] or multi-resolution encoder–decoder networks [43] to a CNN extracting multi-scale features. However, few studies have used 3D semantic segmentation for plant roots, likely owing to the labor-intensive creation of 3D label data and lengthy training of 3D segmentation models. Moreover, methods using artificially synthesized training data have been proposed [44]; however, issues, such as creating synthetic data close to actual root segmentation, hinder their application. Another issue with 3D segmentation is that few applications use rapidly scanned CT volumes with high noise levels. Specifically, in the context of throughput-prioritized crop breeding, the CT volume often exhibits blurring, and the noise level increases owing to the expansion of the scan range and shortening of the scan time [20].

We constructed a non-destructive measurement platform for crop root systems with X-ray CT, mainly for lowland and upland rice cultivars [20, 25, 28]. This platform uses two software, the root system architecture (RSA) visualization software RSAvis3D [20] and RSA vectorizing software RSAtrace3D [25], to measure root traits. RSAvis3D reduces noise levels through 3D median filtering and extracts root features through edge detection, enabling the rapid visualization of crop roots in the soil [20]. RSAtrace3D converts edge-detected images into vector data to extract information, such as root shape, connection, and distribution [25]. In addition, to conduct phenotypic analysis of 3D root distribution for variety development, large pots and rapid CT scanning are required [20]. However, as the pot size increases, the resolution of the CT volume decreases, and the CT volume becomes noisier as the scan time decreases. Therefore, the present study aimed to determine the feasibility of semantic segmentation with rapidly scanned CT volumes of large pots and ascertain the extent to which scan time can be reduced without affecting the results. To this end, we trained the segmentation model with CT volumes obtained at various scan times and compared the results.

## Methods

### Plant materials and cultivation conditions

We used the upland rice cultivar Kinandang Patong. After sterilizing the seed surface using a fungicide, the seeds were soaked in tap water. After 3 days, three seeds were sown in the center of the pots with 20 cm diameter and 25 cm depth, filled with a calcined clay (Profle^®^ Greens Grade^™^, PROFILE Products, Buffalo Grove, IL, USA) as a soil-like root growth substrate. These soils were saturated with 3 × Kimura B hydroponic solution containing 1094.4 µM (NH_4_)_2_SO_4_, 273 µM K_2_SO_4_, 1641 µM MgSO_4_, 549 µM KNO_3_, 1095 µM Ca(NO_3_)_2_, 546 µM KH_2_PO_4_, and 53.4 µM FeC_6_H_5_O_7_·H_2_O. The pH was adjusted to 5.5 using HCl and KOH.

The rice plants were cultivated in a greenhouse under natural light and with 400 W metal–halide lamp irradiation. The duration of the day light was 12 h. The temperature was maintained at 30 °C during the day and 26 °C at night, and humidity at 50%. Tap water was supplied to the bottom of each pot during cultivation.

We prepared two pots: one for model training and the other to evaluate the trained model. The rice plants were grown in different seasons and, therefore, exhibited slight differences in growth. One pot with shallower root systems was used for model training, and lateral roots could not be observed in the CT volume. The other pot with deeper root systems was used for model evaluation, and some lateral roots were observed in the CT volume.

### X-ray CT scan and reconstruction

Four-week-old plants were scanned using an X-ray CT system (InspeXio SMX-225CT FPD HR; Shimadzu Corporation, Kyoto, Japan) with varying scanning times. Details of the scanning conditions are shown in Table 1. Conditions of scan times of 33, 66, 150, 300, 600, and 3600 s are referred to as S0033, S0066, S0150, S0300, S0600, and S3600, respectively. The pots were placed at the center of the CT scanner table. The distance between the CT scanner table center and the X-ray generator was 900 mm, and that between the detector and the generator was 1200 mm. The tube voltage and current were 225 kV and 500 μA, respectively. Cu filters (0.5 mm thickness) were used to harden the X-ray beam. The projection image (1024 × 1024 pixels) was captured using a detector (without binning: 3000 × 3000 pixels at 4.0 frames per second [fps]; with binning: and 1000 × 1000 pixels at 18.0 fps) and computed. The spatial resolution was 0.3 mm. Finally, 860 slices were reconstructed.

**Table 1:** CT scanning times and conditions

Abbreviation	Scanning time (s)	Projection view number	Averaging number	3 × 3 Binning
S3600	3600	1200	12	No
S0600	600	1200	2	No
S0300	300	1200	1	No
S0150	150	600	1	No
S0066	66	1200	1	Yes
S0033	33	600	1	Yes

The scanning time is the time required for scanning only. The time to set the pots and start the equipment is excluded. Binning reduces the scanning time instead of the detector resolution

### Rescaling intensity and cropping of CT volume

The intensity of the CT volume was rescaled from a 16-bit to an 8-bit grayscale. The histogram of pixel intensity exhibited a bimodal distribution under our experimental conditions, with peaks corresponding to air and soil voxels. As rescaling using minimum and maximum values eliminates differences in image intensity when converted to 8-bit, the intensities of the CT volumes were rescaled by setting the peak of the air at 0 and that of the soil at 128. Any values below 0 were replaced with 0, and values exceeding 255 were set to 255. Because values below 0 and over 255 were derived from the air, sand, or mineral particles contaminated in the soil substrates [28], no information was lost in root segmentation. To eliminate regions close to the pot wall, an area with an 9 cm radius from the center of the pot was cropped. This is because roots touching the pot wall changed their direction of elongation, making them less informative for evaluating a variety characteristics [20].

### Image processing with RSAvis3D

An RSA visualization software RSAvis3D was used for extracting root features [20]. RSAvis3D reduced the noise level using 3D median filtering and extracted root features using edge detection. We used RSAvis3D version 1.5 (https://github.com/st707311g/RSAvis3D/, branch 1.5), in which RSAvis3D uses multiple graphics processing units (GPUs) to accelerate extracting roots.

### Generating ground truth data

Ground truth (GT) data were generated using the RSA vectorization software RSAtrace3D [25] version 1.12 (https://github.com/st707311g/RSAtrace3D/, branch 1.12). RSAtrace3D is a GUI-based software that helps users vectorize monocotyledon RSA. The vector data can be converted into the GT volume.

### Volume segmentation and assembling

The CT volume dimensions were 860 (H) × 599 (W) × 599 (D) voxels (Fig. S1a), which were too large for deep learning. Therefore, the CT volume was divided into sub-volumes of 64 (H) × 64 (W) × 64 (D) voxels dimensions (Fig. S1b). Regions at the edges of sub-volumes are less informative; therefore, the results for those regions are generally excluded [45]. Herein, we designated unique and overlapping regions in the sub-volumes (Fig. S1c). Each of the eight voxels at the edges of the sub-volumes was an overlapping region, and the remaining voxels were unique regions. After segmentation prediction, the overlapping regions were deleted, and the unique regions were assembled into the entire RSA segmentation.

### Semantic segmentation model

VoxResNet [46], a deep voxel-wise residual network model typically used for semantic segmentation tasks, was utilized in this study. The model structure was similar to that described previously [46], except that the activation function at the end was changed from softmax to sigmoid, as the number of segmentation classes was only 1: roots. Model training and prediction were performed using PyTorch [47] version 2.0.1 in Python [48] version 3.10.4.

For model training, we used CT volumes S0033, S0066, S0150, S0300, and S0600. All volumes were completely aligned in 3D, as the sample remained fixed on the scan table during sequential scanning and only the imaging conditions were changed. GT was created using the S3600 sample; however, these datasets were not used for model testing. Before model training, the CT volumes (value range: 0–255) were rescaled to 0.0–1.0 by dividing by 255.0, and the GT volumes were converted to Boolean. Each volume was divided into 3042 sub-images. During training, random flips were performed along each axis. Dice loss [49] was used as the loss function to evaluate the spatial overlap between the segmentation results and GT [46]. Adam [50] was used as the optimizer. Given that the learning rate of 0.001 was unstable, a learning rate of 0.0001 was used. The batch size was set to 64, resulting in 48 iterations per epoch. The models were trained for 1500 or 3000 epochs. Training efficiency was determined based on the learning curve of Dice loss and the model weight after 1500 or 3000 epochs. Given that Dice loss exhibited a stable decrease, we judged that the lower the loss after 1500 or 3000 epochs of learning, the better the model performance.

For model testing, using the reconstructed predicted volume and corresponding GT volume, three metrics, namely precision, recall, and Dice were calculated. These metrics were calculated using the following formulas:

1 $$Precision=TruePositive/\left(TruePositive+FalsePositive\right)$$

2 $$Recall=TruePositive/\left(TruePositive+FalseNegative\right)$$

3 $$Dice=2\times Precision\times Recall/\left(Precision+Recall\right)$$

For model evaluation, additional CT volumes were used with the scanning conditions of S0033, S0066, S0150, S0300, and S0600, which were not used for model training. The predictions between scanning conditions were graphically compared.

### Image quality restoration model

The VoxResNet model was trained using the “Noise2Clean” strategy, which involves converting an image with a short scan time to an image with a long scan time. The volumes acquired with short and long scan times were completely aligned in 3D. The last activation function, softmax, was removed because the purpose of this model was image quality restoration, not classification. The mean squared error (MSE) loss [51] was used as the loss function, and Adam [50] was used as the optimizer with a learning rate of 0.00001. The batch size was 64, and the number of iterations per epoch was 48. The model was trained for 500 epochs.

### Volume rendering

Volume data were visualized using the VG Studio MAX 3.2 software (Volume Graphics, Heidelberg, Germany).

### Machine specifications

For image processing and model training, a desktop personal computer (Xeon W-2295 CPU, 96 GB memory, and 3 × NVIDIA GeForce RTX 3090) was used. The operating system was the Ubuntu 20.04.6 LTS.

## Results

### Outline of root segmentation in rapidly scanned CT volumes with high noise levels

**Fig. 1:**
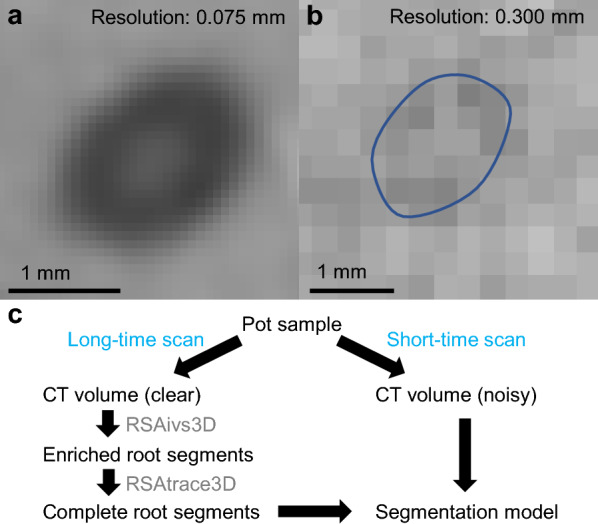
Segmentation model for high-noise and low-resolution root segments in computed tomography (CT) volume. **a** Configuration of root segments in a CT slice. The pot diameter is 9 cm, and the scan time is 10 min. **b** High-noise and low-resolution root segments in a CT slice. The pot diameter is 20 cm, and the scan time is 10 min. The regions with root segments are circled. **c** Outline of obscured root segmentation

Large pot sizes and short scanning times resulted in high noise and low-resolution CT volumes. The root segments scanned from a pot with a 9 cm diameter were easily recognized (Fig. 1a). In contrast, those scanned from a pot with a 20 cm diameter, the maximum size determined by the detector used in this study, were hardly recognizable, with no evident contours (Fig. 1b). Root segmentation was hindered by low obscurity, necessitating developing GT volumes corresponding to these high-noise and low-resolution CT volumes to train the segmentation model. One approach involves scanning the same pot twice, with short and long scan times, and then creating a GT volume from the CT volume with long time scan. Another approach is to use conventional image processing filters to extract root features. Based on these filters, GT images can be created using tracing software [20, 25]. In this study, we implemented both approaches. We scanned the same pot several times with varying scanning times. Subsequently, we generated GT images from the CT volume scanned for a longer duration by tracing the root segments using RSAvis3D and RSAtrace3D software (Fig. 1c). RSAvis3D enriched root features using an edge detection algorithm [20], thereby enabling RSAtrace3D to produce an RSA vector to generate RSA segmentation.

### Scanning time and training efficiency

**Fig. 2:**
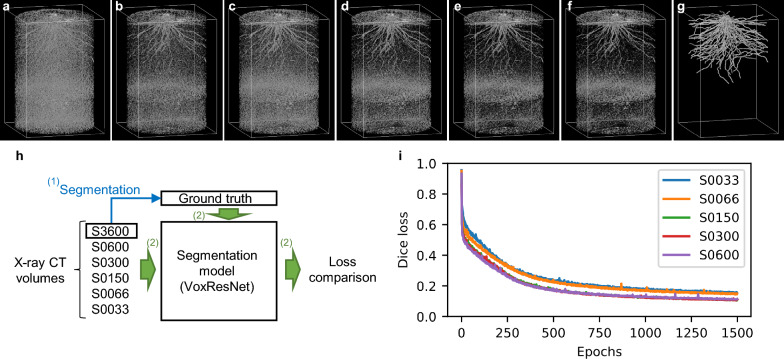
Model training with samples with various scan times. Visualization via RSAvis3D of root distribution of **a** S0033, **b** S0066, **c** S0150, **d** S0300, **e** S0600, and **f** S3600. **g** Ground truth of root segmentation created from S3600. The shape of the bounding box is 25.8 (H) × 18.0 (W) × 18.0 (D) cm. **h** A diagram comparing training efficiency. (1) S3600 was used for segmentation to generate ground truth data. (2) With the ground truth, S0033, S0066, S0150, S0300, and S0600 were used to train the segmentation model. **i** Change in Dice loss for each sample during training

Using the paired dataset of CT and GT volumes, we trained the semantic segmentation models and measured the training efficiency of the segmentation models of S0033, S0066, S0150, S0300, S0600, and S3600. As CT image quality decreases in proportion to scan time when scan time is ≤ 600 s [20], we set six different scan times, adding a scan condition of 3600 s, which exhibits a lower noise level. The edge-detected volumes obtained using RSAvis3D are shown in Fig. 2a–f. Longer scan times tended to result in less noise. The GT volumes were created using the S3600 volume (Fig. 2g), which was paired with each edge-detected volume for model training (Fig. 2h).

For model training, we used VoxResNet, a CNN model of a deep voxel-wise residual network which can improve model performance by increasing the CNN layers. VoxResNet consists of 25 layers and has been widely used for segmentation [46]. We compared the loss changes among datasets of S0033, S0066, S0150, S0300, and S0600 during VoxResNet training (Fig. 2i). Although Dice loss did not fully decrease after training for 1500 epochs, model performance was compared at 1500 epochs because the Dice loss decreased steadily under all conditions. The Dice losses of S0150, S0300, and S0600 were similar (Fig. 2i) at 1500 epochs, indicating that certain noise levels did not affect the training efficiency of the segmentation model. In contrast, the Dice losses of S0033 and S0066 were higher than those at other conditions (Fig. 2i). Notably, these two samples were scanned with binning (Table 1), which substantially affected the training efficiency of the segmentation model. The Dice loss of S0033 was slightly greater than that of S0066 (Fig. 2i). These results indicate that, to a certain degree, noise levels do not affect the training efficiency of VoxResNet.

### Deep learning-based restoration of image qualities before model training

**Fig. 3:**
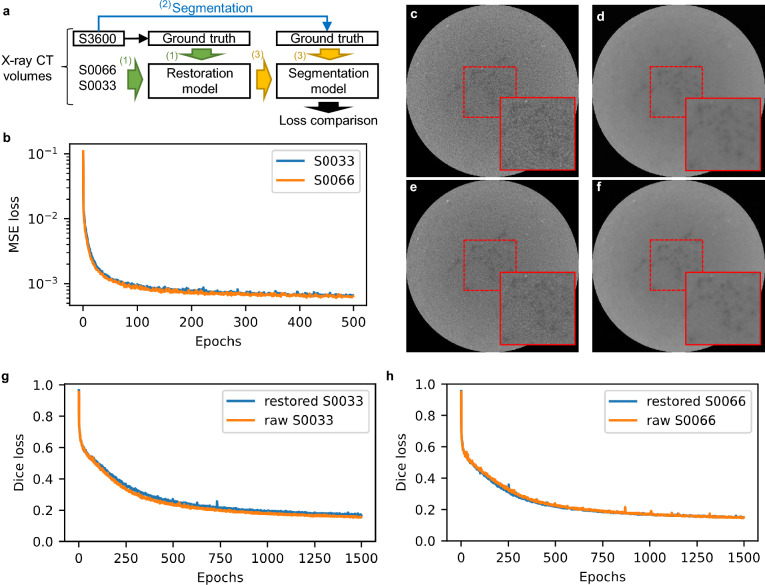
Effects of image quality restoration on training efficiency of segmentation model. **a** A diagram of training of segmentation model combined with image quality restoration. (1) Restoration model was trained with S0033 and S0066 as input data and S3600 as the ground truth. (2) The ground truth of segmentation was generated from S3600. (3) Segmentation models of S0033 and S0066 were trained using the samples with image quality restoration. **b** Change in mean squared error (MSE) loss during training of image quality restoration models. Slices of S0033 (**c**) before and (**d**) after image quality restoration. Slices of S0066 (**e**) before and (**f**) after image quality restoration. The area enclosed by the red dashed line is enlarged. Changes in Dice losses of (**g**) S0033 and (**h**) S0066 during segmentation training with and without image quality restoration

Assuming that the reduced segmentation efficiency in volumes with a shorter scan time was caused by image quality degradation, restoring image quality can help increase segmentation efficiency in volumes with a shorter scan time. To test this hypothesis, we trained VoxResNet as an image quality restoration model; the volumes of S0033 and S0066 were used as input data, and the volume of S3600 as the GT (Fig. 3a). After training 500 epochs, the MSE losses for S0033 and S0066 were approximately 0.006 (Fig. 3b). The noise levels of CT slices were apparently reduced (Fig. 3c–f), and the peak signal-to-noise ratio of S0033 and S0066 were 28.27 and 28.90 before restoration and 31.80 and 31.94 after restoration, respectively. These results indicate that the image qualities of S0033 and S0066 were restored. Using samples with and without image-quality restoration, we trained a segmentation model and compared the Dice loss changes of S0033 (Fig. 3g) and S0066 (Fig. 3h) over 1500 epochs. No differences were observed in the Dice losses between samples with and without image-quality restoration. These findings indicate that, at least in this case, deep learning-based image quality restoration is ineffective in increasing the training efficiency of the segmentation model.

### Preprocessing before root segmentation

**Fig. 4:**
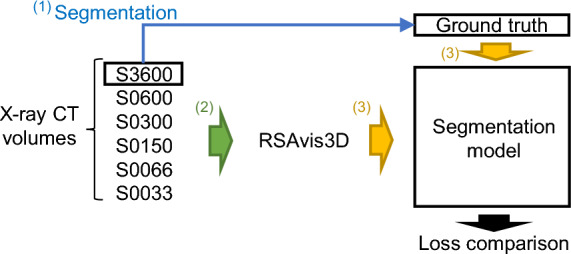
A diagram of segmentation model training combined with RSAvis3D. (1) The ground truth of segmentation was created from S3600. (2) The CT volumes were filtered using RSAvis3D. (3) VoxResNet segmentation models were trained with S0033, S0066, S0150, S0300, S0600, and S3600

Assuming that prefiltering image processing, not based on deep learning, increases the training efficiency of the segmentation model, we trained two types of segmentation models. One used the CT volume, and the other used the preprocessed CT volume using RSAvis3D. Subsequently, we compared the Dice loss at 1500 epochs (Fig. 4a). The results revealed that preprocessing improved the Dice loss by approximately 10% (Table 2). No cases of reduced efficiency were observed, indicating that the feature extraction of RSAvis3D, which is not based on deep learning, effectively improved the training efficiency of the segmentation model.

**Table 2:** Effect of preprocessing on Dice loss of the training dataset

Sample	Without preprocessing	With preprocessing	Improvement (%)
S0033	0.154	0.142	7.7
S0066	0.150	0.126	15.8
S0150	0.123	0.112	8.8
S0300	0.114	0.107	5.8
S0600	0.115	0.100	13.2
S3600	0.110	0.099	10.1

Assuming that the value of “Without processing” is A and the value of “With processing” is B, “Improvement” was calculated by (A–B)/A

To evaluate the effects of preprocessing, we calculated three model metrics, namely precision, recall, and the Dice values. Metrics were computed using the entire reconstructed 3D volumes, rather than segmented sub-blocks. Our findings revealed that preprocessing improved all metrics for almost all samples, with a particularly notable increase in precision (Table 3). This result suggested that preprocessing increased segmentation metrics by reducing false positives or increasing true positives. In addition, the precision of S0066 and S0600 without processing was lower than that of other samples, suggesting that preprocessing increased the stability of model training. Using the model trained with preprocessing and CT volumes used for model training, root segmentation was predicted (Fig. S2), with nearly all the root segments accurately identified.

**Table 3:** Effect of preprocessing on metrics of the training dataset

Condition	Precision	Recall	Dice
S0033	0.822–0.814 (0.008)	0.885–0.879 (0.006)	0.853–0.846 (0.007)
S0066	0.868–0.748 (0.120)	0.896–0.839 (0.057)	0.882–0.791 (0.091)
S0150	0.883–0.839 (0.044)	0.896–0.881 (0.015)	0.889–0.858 (0.031)
S0300	0.881–0.861 (0.020)	0.905–0.908 (− 0.003)	0.893–0.884 (0.009)
S0600	0.917–0.788 (0.129)	0.888–0.872 (0.061)	0.902–0.828 (0.074)
S3600	0.895–0.892 (0.003)	0.924–0.904 (0.020)	0.909–0.898 (0.011)

Assuming that the metric value with processing is A and the value without processing is B, the table is presented as A—B (difference)

**Fig. 5:**
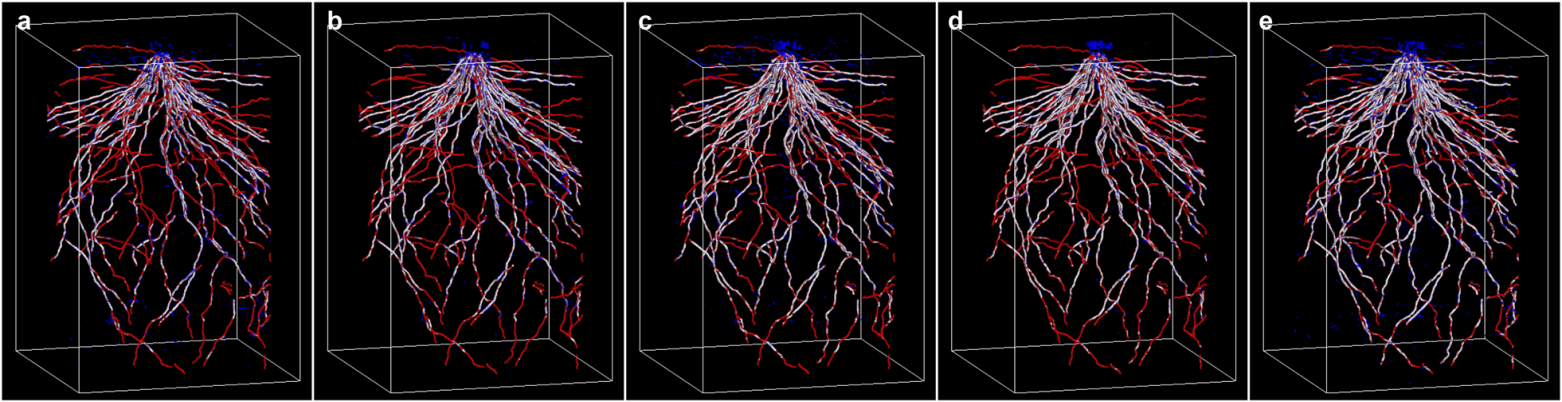
Comparison of segmentation results with ground truth. Predicted segmentation of (**a**) S0033, (**b**) S0066, (**c**) S0150, (**d**) S0300, and (**e**) S0600. White: predicted segments overlapping the ground truth, blue: predicted segments not overlapping the ground truth, red: ground truth segments not predicted. The shape of the bounding box is 25.8 (H) × 18.0 (W) × 18.0 (D) cm

To validate the models, we additionally cultivated a rice plant and scanned its pots to prepare CT volumes that were not used for model training. Scanning conditions were S0033, S0066, S0150, S0300, and S0600. False positives (blue) were noted in all conditions at similar frequencies (Fig. 5), whereas false negatives (red) were more frequently observed in S0033. False positives were observed in the shoot area, near the ground surface, and on the surface of the true segments (white). This result is presumably attributed to limited shoot data in the training data, numerous voids due to incompletely packed soil particles near the ground surface, and incomplete GT data due to high-noise and low-resolution CT volumes. Almost all false negatives observed under all conditions appeared to be lateral roots, likely because the training data contained few lateral roots. In addition, we calculated the precision, recall, and Dice of the reconstructed prediction volumes and found that all metrics were lower than those of the training datasets, particularly in S0033 and S0066 (Table 4). These results indicate that more training datasets with scan times ≥ 150 s should be used to train the model.

**Table 4:** The metrics of the validation dataset

Condition	Precision	Recall	Dice
S0033	0.377	0.592	0.461
S0066	0.419	0.634	0.505
S0150	0.516	0.685	0.589
S0300	0.468	0.688	0.557
S0600	0.558	0.609	0.583

### High-precision models that can be developed based on our trials

**Fig. 6:**
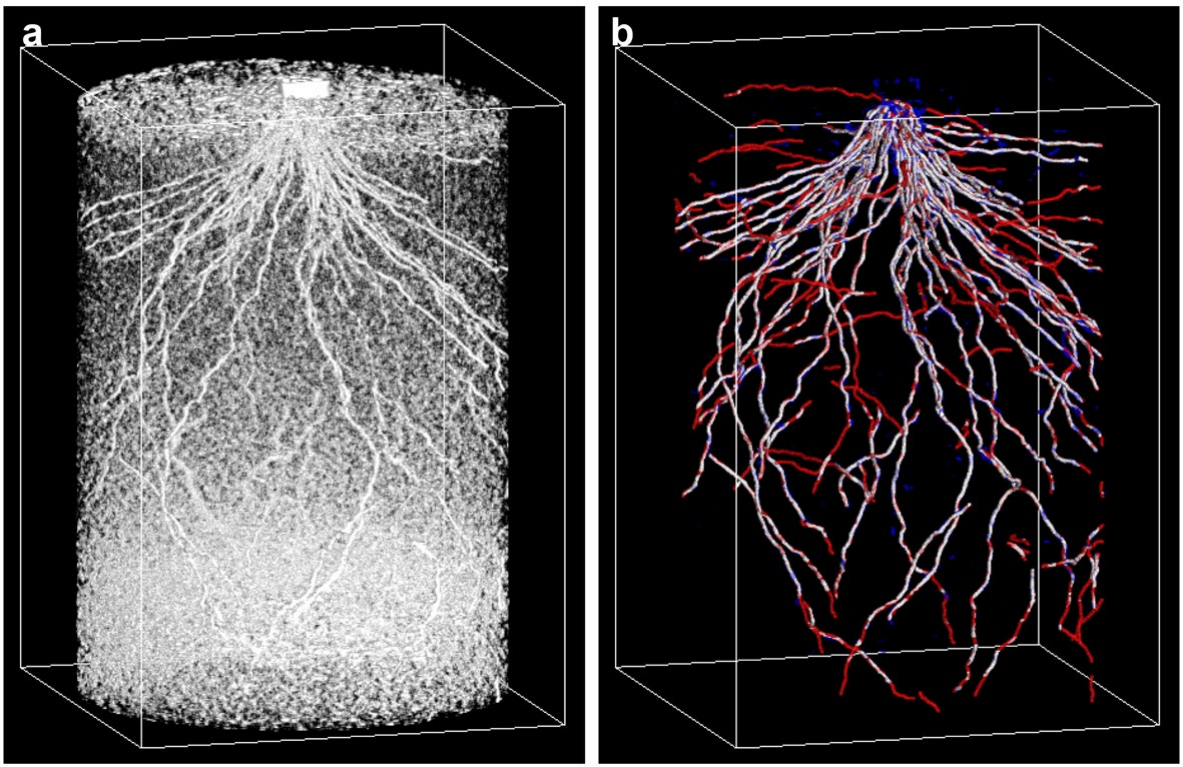
High-precision segmentation model. **a** RSAvis3D-filtered volume of S0150. **b** Predicted segmentation. White: predicted segments overlapping the ground truth, blue: predicted segments not overlapping the ground truth, red: ground truth segments not predicted. The shape of the bounding box is 25.8 (H) × 18.0 (W) × 18.0 (D) cm

To assess the accuracy of the root prediction models, we trained the models over a greater number of epochs, as the Dice loss did not decrease completely at 1500 epochs (Fig. 2i). In addition, we trained the model with CT and RSAvis3D-processed volumes, given that RSAvis3D may eliminate some features that appeared only in the original CT volumes. After 3000 epochs of training for S0150, the Dice loss was 0.093, a 17% improvement compared to the loss observed at 1500 epochs with only RSAvis3D-processed volumes. Using this model, we predicted root segments using S0150 for model evaluating (Fig. 6, Movies S1 and S2). The false-negative (red) segments were similar to those predicted by the model trained for 1500 epochs with only RSAvis3D-processed images; however, many false-positive segments disappeared (Fig. 6b). These results indicate that false positives can be reduced by increasing epochs or including original CT volumes or both and that false negatives cannot be reduced. In other words, roots with features absent in the training data were identified as false negatives in the prediction data.

## Discussion

Semantic segmentation is a powerful tool for extracting ROIs from images with complex backgrounds. In the present study, we demonstrated the feasibility of root segmentation in rapidly scanned CT volumes with high noise levels. The approach involved creating a pair of CT volumes with short and long scan times, generating GT volumes from the CT volumes with a long scan time, and training the model with the GT and CT volumes with a short scan time. In addition, using a pot with a 20 cm diameter, we demonstrated that reducing the scan time to 150 s had minimal effect on the segmentation results. However, some roots were not predicted by segmentation, mainly because these were lateral roots not included in the training data. This limitation can be addressed by expanding the training data. Currently, the demand for creating semantic segmentation models to isolate ROIs from high-noise and low-resolution images is high. In the medical field, semantic segmentation is used to isolate organs to accurately diagnose diseases [46, 52]; however, shortening the scanning time is necessary to minimize exposure [51, 53–55]. Furthermore, owing to limitations in equipment performance, only low-quality images are frequently available. We believe our findings will contribute to addressing these challenges.

In the present study, we observed no differences in the model training efficiency between S0150, S0300, and S0600, indicating that the semantic segmentation model can be used for high-throughput and low-dose root phenotyping. We required 1 min for RSAvis3D preprocessing and 5 days for model training. Thus, model training is the bottleneck of this method, and using fewer GPUs requires an extended training duration. Moreover, the model was trained at a constant learning rate, and introducing a learning rate scheduler could potentially shorten the training time. As the optimal learning rate depends on the soil type and plant species in the CT volumes, examining the learning rate when training the model under new conditions is necessary. Once the model training is complete, model prediction can be accomplished in only a few minutes, which is sufficiently rapid. This high-throughput technique can be used for genetic analysis requiring multiple samples. In addition, CT imaging is non-destructive and can measure changes over time; however, X-ray exposure must be considered when imaging the same individual repeatedly [20, 29]. Therefore, the segmentation model used in this study is advantageous, as shorter scan times allow for more frequent imaging of the same individual.

The minimum number of volumes required for the method presented in our study is two: one for model training and the other for model evaluating, indicating that only two GT data sets should be generated. Generally, segmentation model training requires less training data than other model training, such as classifiers. For example, in 2D, root prediction in the rhizobox uses 50 images [33], and in the trench profile, the surface with exposed roots in trenches dug near the plants utilizes 30 images [38]. In contrast, approximately 50,000 images are used to train a classification model for plant diseases [56]. This discrepancy arises because semantic segmentation can address a few classes, such as root segmentation, and it allows for the subdivision of one image into several images. In the trench profile, ten images are divided into 360 images for model training [38]. In the segmentation model in the present study, one CT volume was divided into 3042 sub-volumes. Therefore, if a new segmentation model of the X-ray CT volume is trained, at least one (or two for evaluation) dataset pairs of CT and GT volumes are required. Using more data sets for training will result in a more generalized model and mitigate potential biases in the data sets used for training, such as the lack of lateral root segmentation in this study.

In addition, image quality restoration before segmentation for increasing training efficiency did not increase training efficiency. This result may be because we used the same CNN structure in the restoration and segmentation models. Segmentation accuracy of degraded images, including reduced size, high noise levels, and blur, increases using image restoration based on a generative adversarial network [57]. Moreover, pretraining the decoder in the CNN structure is effective for few-shot semantic segmentation [58], indicating that pre-segmentation image quality restoration is effective. We postulated that a model with a different structure than the image quality restoration model used in this study may improve segmentation accuracy. Although image quality restoration did not affect the learning efficiency globally, the efficiency may have varied locally. However, as the objective of this study was to increase global learning efficiency reduced by shortened scan times, a more detailed analysis was not performed. Moreover, Dice losses during 1500 epochs of segmentation model training in S0150, S0300, and S0600 were similar despite different noise levels, indicating that this noise level does not affect the segmentation results using VoxResNet.

In the present study, the RSAvis3D preprocessing improved the model learning efficiency by approximately 10%. The main RSAvis3D algorithms are 3D median filtering and edge detection [20]. Some semantic segmentation models use median filtering [59, 60] and edge detection [61, 62] to increase segmentation accuracy, implying that 3D median filtering simplifies the complexity of the image texture, while edge detection focuses on the information of root features. In this study, the training efficiency of the segmentation model was robust at certain noise levels. These results suggest that edge detection plays a role in increasing training efficiency; however, we could not elucidate whether 3D median filtering or edge detection was more effective.

The model in this study was trained for rice roots. However, we need to consider a few conditions when applying it to other crops. First, the proportion of classes in the training data should be determined. Rice RSAs are considerably simple, mainly consisting of adventitious roots with lateral roots. In contrast, the RSAs of legume crops, such as soybean (*Glycine max*), are more complex, with seminal and lateral roots with nodules. Therefore, at least two classes, root and nodule, are required. Generally, the proportion of nodule to root voxels is considerably less than 1 (possibly zero for individuals without nodules), indicating imbalanced training data, which is a limitation for proper training [63]. GTs should be created from multiple CT volumes to ensure adequate training data for the nodules, and the extremely imbalanced training data set should be adjusted to address these issues. Furthermore, this study only created segmentations and did not evaluate traits. As the segmentation is just an image, computing the RSA traits by vectorization or other methods is necessary [64]. Moreover, given that segmentation is error-prone, segmentation-free image analysis techniques have been developed [65]. However, segmentation can also be used as a preprocessing step to extract ROIs from images with complex backgrounds. For example, segmentation can be performed with this method on a noisy image scanned at high speed and combined with another method that computes some features from the resulting root system image. This study contributes to advancing root research using image analysis and root phenomics.

## Conclusions

In the present study, the semantic segmentation model was trained for root segmentation in high-noise and low-resolution CT volumes. The trained segmentation model can be used for root segmentation in 5 min, including scanning, image processing, and segmentation prediction. This study facilitates root system analysis using CT imaging and analysis of high-noise and low-resolution images, such as medical images created by low-dose scanning.

### Supplementary Information

Supplementary Material 1: Fig. S1 Dividing CT volume for the training of semantic segmentation. (a) CT volume before division. (b) CT volume divided into small sub-volumes. (c) The sub-volume comprising unique and overlapped regions.

Supplementary Material 2: Fig. S2 Segmentation results of CT volume used for training. Predicted segmentation of (a) S0033, (b) S0066, (c) S0150, (d) S0300, and (e) S0600.

Supplementary Material 3: Movie S1 Animation of 3D rendering of RSAvis3D-processed S0150.

Supplementary Material 4: Movie S2 Animation of 3D rendering of predicted S0150. White: predicted segments overlapping the ground truth, blue: predicted segments not overlapping the ground truth, red: ground truth segments not predicted.

## Data Availability

The scripts for the training and prediction of the 3D semantic segmentation are available at the GitHub repository (https://github.com/st707311g/SStrainer3D/, branch 1.0). The datasets used during the current study are available from the corresponding author upon reasonable request.

## Abbreviations

3D: Three-dimensional
CT: Computed tomography
fps: Frames per second
GPU: Graphics processing unit
GT: Ground truth
MSE: Mean squared error
ROI: Regions of interest
RSA: Root system architecture
