# Supplementary figures and images for: Convolutional neural networks combined with conventional filtering to semantically segment plant roots in rapidly scanned X-ray computed tomography volumes with high noise levels
Source: Plant Methods. 2024 May 21;20:73. doi: 10.1186/s13007-024-01208-0 (PMC11106967; doi:10.1186/s13007-024-01208-0)

## Slide 1
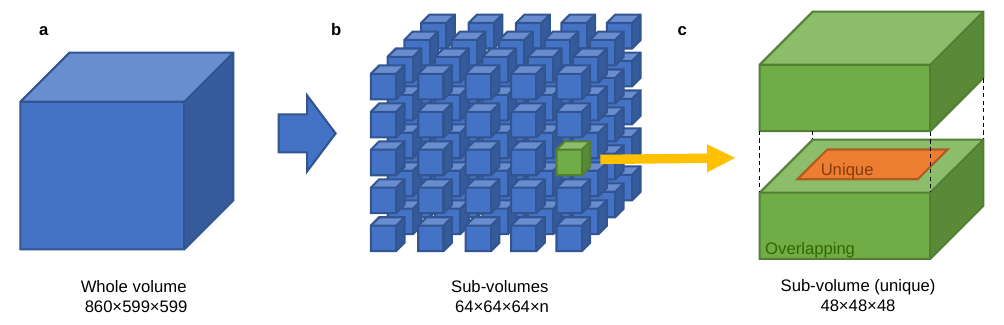

a
b
c
Unique
Overlapping
Sub-volume (unique)
48×48×48
Whole volume
860×599×599
Sub-volumes
64×64×64×n

Supplement: Supplementary file 1 — Supplementary Material 1: Fig. S1 Dividing CT volume for the training of semantic segmentation. (a) CT volume before division. (b) CT volume divided into small sub-volumes. (c) The sub-volume comprising unique and overlapped regions. [file 13007_2024_1208_MOESM1_ESM.pptx]

## Slide 1
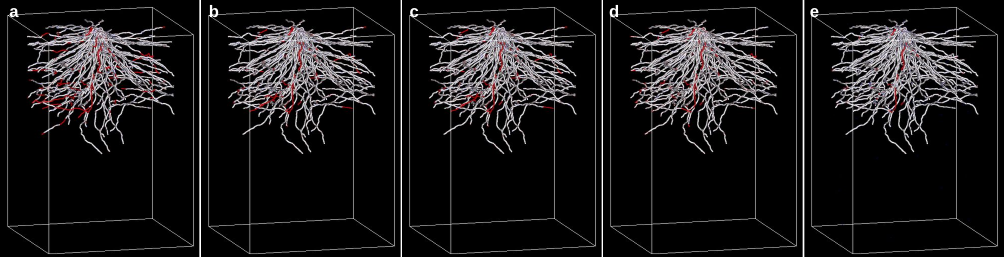

a
b
c
d
e

Supplement: Supplementary file 2 — Supplementary Material 2: Fig. S2 Segmentation results of CT volume used for training. Predicted segmentation of (a) S0033, (b) S0066, (c) S0150, (d) S0300, and (e) S0600. [file 13007_2024_1208_MOESM2_ESM.pptx]
